# Long Non-coding RNA in Plants in the Era of Reference Sequences

**DOI:** 10.3389/fpls.2020.00276

**Published:** 2020-03-12

**Authors:** Hikmet Budak, Sezgi Biyiklioglu Kaya, Halise Busra Cagirici

**Affiliations:** ^1^Montana BioAgriculture, Inc., Bozeman, MT, United States; ^2^Engineering and Natural Sciences, Molecular Biology, Genetics and Bioengineering Program, Sabancı University, Istanbul, Turkey

**Keywords:** wheat, barley, whole genome sequencing, computational identification, long non-coding RNA

## Abstract

The discovery of non-coding RNAs (ncRNAs), and the subsequent elucidation of their functional roles, was largely delayed due to the misidentification of non-protein-coding parts of DNA as “junk DNA,” which forced ncRNAs into the shadows of their protein-coding counterparts. However, over the past decade, insight into the important regulatory roles of ncRNAs has led to rapid progress in their identification and characterization. Of the different types of ncRNAs, long non-coding RNAs (lncRNAs), has attracted considerable attention due to their mRNA-like structures and gene regulatory functions in plant stress responses. While RNA sequencing has been commonly used for mining lncRNAs, a lack of widespread conservation at the sequence level in addition to relatively low and highly tissue-specific expression patterns challenges high-throughput *in silico* identification approaches. The complex folding characteristics of lncRNA molecules also complicate target predictions, as the knowledge about the interaction interfaces between lncRNAs and potential targets is insufficient. Progress in characterizing lncRNAs and their targets from different species may hold the key to efficient identification of this class of ncRNAs from transcriptomic and potentially genomic resources. In wheat and barley, two of the most important crops, the knowledge about lncRNAs is very limited. However, recently published high-quality genomes of these crops are considered as promising resources for the identification of not only lncRNAs, but any class of molecules. Considering the increasing demand for food, these resources should be used efficiently to discover molecular mechanisms lying behind development and a/biotic stress responses. As our understanding of lncRNAs expands, interactions among ncRNA classes, as well as interactions with the coding sequences, will likely define novel functional networks that may be modulated for crop improvement.

## Introduction

Since the realization of regulatory information contained within the non-protein-coding parts of DNA, efforts to identify non-coding RNA molecules have greatly accelerated. Advances in RNA sequencing technology have contributed to this acceleration and the discovery of non-coding RNAs, including lncRNAs, which elucidated their structures and functions. As our understanding of the regulatory roles of lncRNAs has improved, the importance of these non-coding molecules has become more apparent. However, there is still much to discover about the functions of lncRNAs in cellular pathways.

A step further to understand both coding and non-coding elements was taken recently for wheat and barley: high-quality reference sequences have been published (Mascher et al., 2017; IWGSC, 2018). Wheat and barley are two of the most consumed and cultivated crops; thus, increasing the yield have been the ultimate goal for breeders and scientists to overcome the effects of population growth and climate change. Having a reference genome in hand improved the accuracy of the analyzes to find the origins of favorable traits and regulatory mechanisms that control the expression of the genes responsible for those traits. Therefore, wheat and barley reference sequences have opened a new era in the field of multi-omics research, allowing more accuracy and robustness toward the lightening of the undiscovered mechanisms within these important crops.

## Biogenesis of lncRNAs

Long non-coding RNAs (lncRNAs) are defined as transcripts longer than 200 bp that cannot construct a full-length protein (Kapranov et al., 2007). The lack of discernable coding potential is what mainly differentiates lncRNAs from mRNAs.

Similar to mRNAs, most lncRNAs are transcribed by RNA polymerase II and are subject to 5′-end capping, alternative splicing, and the addition of 3′ poly-A tails (Chekanova, 2015). Plant lncRNAs can be transcribed by two additional polymerases; RNA Pol IV or RNA Pol V (Wierzbicki et al., 2008). Unlike Pol II transcripts, these lncRNAs are less characterized and possess some structural differences such as lack of poly-A tails (Zhou and Law, 2015). Identification of RNA Pol IV or PolV transcribed lncRNAs is particularly challenging due to their extremely low expression and instability (Rai et al., 2018). However, these transcripts are the major players driving RNA-mediated DNA methylation (RdDM). Plants have evolved a highly sophisticated RNA interference-dependent RdDM mechanism to ensure genomic stability (Matzke and Mosher, 2014). Briefly, in this pathway, an lncRNA transcribed by RNA polymerase IV is later processed into 24-nt small interfering RNAs (siRNAs) (You et al., 2013). lncRNA transcribed by RNA polymerase V is recognized by the siRNA-AGO complex and drives this complex to the chromatin target site together with chromatin modifying enzymes. Following interaction with the AGO complex, additional proteins and methyltransferases are recruited to cytosine residues at the target region to initiate gene silencing (Wierzbicki et al., 2008).

RNA polymerase IV transcripts reportedly act mostly as siRNA precursors, whereas RNA polymerase V and some RNA polymerase II transcripts are sRNA targets. RNA polymerase IV and V transcripts have mostly been studied in *Arabidopsis thaliana*, where a recent study identified 10s of 1000s of RNA polymerase IV-dependent lncRNAs using an RNA polymerase IV mutant (Li et al., 2015).

## Influence of RNA Sequencing Technologies on the Discovery of lncRNAs

**FIGURE 1:**
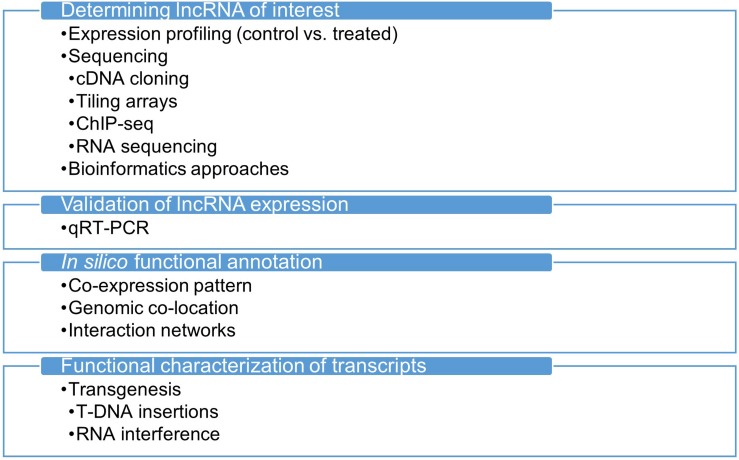
A general workflow for the identification and characterization of transcripts.

A general method for identifying and functionally characterizing transcripts is shown in Figure 1. Improvements in RNA sequencing technology paved the way for expanding our understanding of RNA. Previous attempts to uncover transcriptomes relied mostly on microarray technology, which is inefficient and limited in coverage of the whole transcriptome, whereas next-generation DNA and RNA sequencing applications are readily available on many platforms, offering better and more consistent quality (Denoeud et al., 2008; Ozsolak and Milos, 2011). Together with the development of computational tools, the most striking and unexpected evidence has been collected from the non-coding parts of the genome, revealing the transcription of numerous non-coding RNA molecules in various structures and roles. Of all the RNA species discovered to date, lncRNAs are the most unclear class of molecules and might still hide many unknown features. To reveal the secrets of lncRNAs and other non-coding RNA species, new RNA sequencing applications have been developed. For example, while conventional RNA sequencing allowed sequencing of up to 600 nucleotides at a time, the deep sequencing approach has enabled sequencing of longer reads at high accuracy (Malone and Oliver, 2011; Chu et al., 2015). RNA capture sequencing detects targeted RNA molecules with low abundance in the transcriptome (Mercer et al., 2011; Clark et al., 2015), and was designed to overcome obstacles in conventional RNA sequencing in detecting low-abundance lncRNAs.

To study the functions of lncRNAs, several immunoprecipitation-based methods have been developed that reveal the interacting RNA partners of specific proteins, together with high-throughput sequencing. ChIRP-seq is one of these methods, and involves precipitation of *in vivo* cross-linked RNA–DNA and RNA–protein hybrids by a biotin-streptavidin interaction and then sequencing of the RNA and DNA molecules that appear in the precipitated hybrids (Chu et al., 2011, 2015). CLIP-seq is another immunoprecipitation-based technique that has been used to explore miRNA–lncRNA interactions (Murigneux et al., 2013; Li J. et al., 2014). As demonstrated by these examples, RNA sequencing technology can be improved and modified according to the needs of the study. Improvements in the efficiency and accuracy of RNA and DNA sequencing techniques, not only for the identification of lncRNAs, but also for other RNA species, will lead to a more complete understanding of the secrets of the cellular mechanisms and their regulators.

*In silico* predictions based on sequencing have revealed many lncRNAs with expression patterns that remain to be confirmed. qRT-PCR allows the detection and quantification of expression in real time and is therefore widely used to verify the expression of *in silico*-predicted lncRNAs (Shuai et al., 2014). lncRNAs have been functionally annotated based on co-expression patterns, interaction networks, or both. Functions of lncRNAs can be predicted based on co-expressed protein-coding genes and/or genomic co-localization of genes (Guttman et al., 2009; Liao et al., 2011). For example, the lncRNAs COOLAIR and COLDAIR are expressed at the *FLC* locus and control *FLC* expression (Heo and Sung, 2011). Moreover, lncRNAs can serve as sRNA targets, preventing interaction between the sRNA and its protein-coding target, thereby enhancing the function of a particular protein-coding gene (Britton et al., 2014; Shuai et al., 2014).

These interaction networks between lncRNA, miRNA, and mRNAs suggest that some lncRNAs function as endogenous target mimics (Franco-Zorrilla et al., 2007; Chen et al., 2013). lncRNAs can also serve as sRNA precursors, with the downstream patterns of the corresponding sRNA revealing the involvement of lncRNAs in various molecular pathways (Matzke and Mosher, 2014; Ariel et al., 2015). Potential functions of lncRNAs can be confirmed by construction of trangenic lines with either downregulation or overexpression of genes. T-DNA insertions can be used for either gain-of-function or loss-of-function mutagenesis (Radhamony et al., 2005) whereas RNAi interference results in loss-of-function. For example, Zhu et al. (2014) identified lncRNAs in *Arabidopsis thaliana* that were differentially expressed during infection with *Fusarium oxysporum* and confirmed antifungal activity of 10 lincRNAs using T-DNA insertion and RNAi lines. Identification and confirmation of the interactions and functions of these non-coding RNAs is critical for the characterization of important molecular pathways.

## lncRNA Annotation From RNA Sequencing Data

When using RNA sequencing data to annotate lncRNAs, computational procedures commonly begin with the alignment of sequencing reads on the reference genome, if available, and the assembly of transcript models from the mapped reads using computational tools that can be chosen from a wide range of software and algorithms based on their features and computational requirements (Ilott and Ponting, 2013). When a reference genome is lacking for the species of interest, the assembly can be accomplished *de novo* although this strategy is more error-prone by being more sensitive to sequencing errors and chimeric molecules, and requiring more coverage in sequencing (Martin and Wang, 2011). After this point, the assembled transcripts should be evaluated to distinguish lncRNAs from a variety of non-coding RNAs and protein-coding mRNAs. Although complex and unclear features of lncRNAs have led researchers to adopt different methods and tools for the identification process, they seem to agree on a few basic criteria to select lncRNAs from other RNAs, such as minimum length. Many studies assume a 200-nucleotide length threshold to separate lncRNAs from snRNAs. Even though the presence of lncRNAs below this threshold has not been fully disproven, it is useful to eliminate snRNAs from the data (Ma et al., 2013). However, this criterion is mostly arbitrary and, alone, cannot define lncRNAs. In addition, this criterion does not distinguish between lncRNAs and mRNAs, since both types of RNA are commonly longer than 200 nucleotides (Milligan and Lipovich, 2015).

Therefore, for sequences that pass the first criterion, researchers usually assess open reading frame (ORF) content and length. Since transcripts containing long ORFs are assumed to be translated into full-length proteins, lncRNAs are expected to lack an ORF, or at least a long ORF (Boerner and McGinnis, 2012). Previous studies have speculated that most lncRNAs contain a short ORF (Banfai et al., 2012; Lv et al., 2013; Ruiz-Orera et al., 2014) and can occupy ribosomes, with contradictory conclusions about whether they encode protein products (Guttman et al., 2013; Ruiz-Orera et al., 2014; Popa et al., 2016). Despite lacking a clear explanation of the translational features of lncRNAs, these conflicting findings agree on another arbitrarily determined criterion, that is, an ORF size threshold of encoding 100 amino acids (Ilott and Ponting, 2013; Musacchia et al., 2015). After eliminating transcripts containing ORFs above the threshold, transcripts that satisfy the ORF size criterion are often examined to determine whether the remaining ORFs potentially encode any functional proteins. Several methods are used to calculate coding potentials and various algorithms can be used to assess candidate transcripts in terms of ORF presence, quality, intactness, and similarities to sequences encoding known proteins (Boerner and McGinnis, 2012; Mattick and Rinn, 2015). As this step is highly dependent on the quality of RNA sequencing reads and alignments on reference genomes, low-quality sequencing or alignment data, or lack of a reference genome, increases the chances of misleading coding potential calculations.

The use of machine learning techniques alone has increased the accuracy of coding potential calculations to over 90% (Kong et al., 2007; Hoff and Stanke, 2013; Sun et al., 2013). Nonetheless, due to slight differences in the approaches of conventional coding potential calculation tools, combining several of these tools may increase the stringency of the identification pipeline (Pauli et al., 2012).

The final criterion applied in many lncRNA identification pipelines involves exclusion of candidate transcripts that exhibit homology to known coding sequences, proteins, or protein domains. Similar to coding potential, homology can be assessed by several methods that use different databases for transcript comparisons (Jia et al., 2010; Pauli et al., 2012). However, a caveat of this criterion is the loss of these exonic lncRNAs, leaving only lncRNAs expressed from intronic or intergenic spaces that do not overlap with the exons of any protein-coding genes (Housman and Ulitsky, 2016). Therefore, a balance between sensitivity and robustness must be properly maintained while designing the pipeline with elimination thresholds tailored to the aim of the study.

## Structural and Functional Characterization of lncRNAs

**FIGURE 2:**
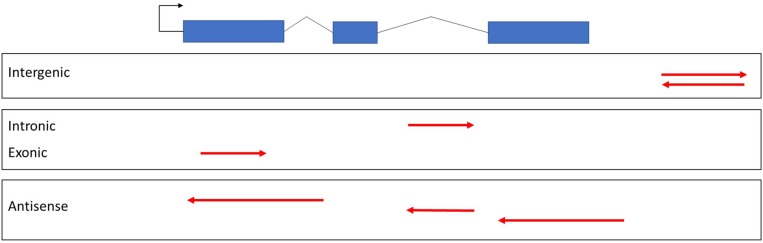
Classification of lncRNAs. Exons of protein coding genes were shown in blue bars. Red arrows indicate lncRNAs based on genomic location and direction of transcription with respect to protein coding gene.

lncRNAs can be classified with respect to their genomic location and the direction of transcription (Figure 2), including intergenic, intronic, or exonic regions in the sense and antisense directions (Mattick and Rinn, 2015). The most controversial class was exonic lncRNAs that transcribed in the sense orientation. The lncRNA transcripts intersecting with the exons of protein coding genes had been eliminated until the latest release of GENCODE v7 catalog of human long non-coding RNAs (Derrien et al., 2012). However, some non-coding transcripts may arise from alternative splicing or truncation of first or last exons of protein coding genes. For example, SRA1 gene encodes for a lncRNA transcript [steroid receptor RNA activator (SRA)] as well as a protein coding transcript (SRAP) by alternative splicing (Sheng et al., 2018). Functional characterization of SRA have been performed well in both human and mouse (Nam et al., 2016). In fact, functions of SRAP has been less studied when compared to SRA. Although, there are currently not exonic lncRNAs with known functions available in plants yet, exonic lncRNAs have been reported in several plant species but without functional characterization (Liu et al., 2013; Quattro et al., 2017). Broadly, plant lncRNAs with known functions are classified as long intergenic non-coding RNAs (lincRNAs), intronic non-coding RNAs (incRNAs), and natural antisense transcripts (NATs) (Table 1).

**TABLE 1:** Examples of lncRNAs with known functions in plants.

**lncRNAs**	**Species**	**Class**	**Biological function**	**Mechanism of action**	**Reference**
**Plant lncRNAs with best-studied functions**					
COOLAIR/COLDAIR	*A. thaliana B. distachyon*	NATs/incRNAs	Mediate flowering process	Histone modification	Heo and Sung, 2011; Csorba et al., 2014; Shafiq et al., 2015
APOLO	*A. thaliana*	lincRNAs	Modulates polar auxin transport	Chromatin-loop formation	Ariel et al., 2014
LDMAR	*Rice*	lincRNAs	Regulates photoperiod-sensitive male sterility	RNA-dependent DNA methylation (RdDM)	Ding et al., 2012
IPS1	*A. thaliana*	lincRNAs	Balances phosphate homeostasis	Endogenous target mimicry	Franco-Zorrilla et al., 2007
HID1	*A. thaliana*	NATs	Enhances photomorphogenesis	Chromatin association	Wang et al., 2014
Enod40	*Medicago*, rice, maize, legumes, *A. thaliana, * soybean	lincRNAs	Regulates nodulation	Protein relocalization	Yang et al., 1993; Rohrig et al., 2002; Campalans, 2004; Gultyaev and Roussis, 2007
**Plant lncRNAs with less **characterized** functions**					
WSGAR	Wheat	N/K*	Modulates seed germination	sRNA target and sRNA precursor	Guo et al., 2018
HvCesA6	Barley	NATs	Regulates cell wall synthesis	sRNA precursor	Held et al., 2008
***cis-***NAT PHO1;2	Rice	NATs	Phosphate homeostasis and plant fitness	Translation enhancer	Jabnoune et al., 2013
LAIR	Rice	NATs	Increases grain yield	Uncharacterized	Wang et al., 2018
**Twisted lead (TL)**	Rice	NATs	Regulates leaf morphology development	Chromotin modifications	Liu et al., 2018

*N/K: not known.

lncRNAs transcribed outside of protein-coding genes are loosely classified as lincRNAs. Most research on plant lncRNAs has focused on lincRNAs, leading to the identification of several lncRNAs with well-studied functions, such as LDMAR (Ding et al., 2012), APOLO (Ariel et al., 2014), IPS1 (Franco-Zorrilla et al., 2007), and Enod40 (Campalans, 2004). lncRNAs transcribed from intronic regions in the sense direction are called incRNAs. COLDAIR, transcribed from the first intron of *Flowering Locus C* (*FLC*), is the best-known plant incRNA (Heo and Sung, 2011). lncRNAs transcribed from the antisense direction to a protein-coding gene are classified as NATs. Well-studied examples of plant NATs include COOLAIR (Csorba et al., 2014) and HID1 (Wang et al., 2014). Recently, an antisense transcript of HvCesA6, which acts as a precursor to small RNA (sRNA) targeting the CesA6 gene, was shown to be involved in regulating cell wall synthesis in barley (*Hordeum vulgare*) (Held et al., 2008). Several plant NATs with newly characterized functions include *cis-*NAT PHO1;2 (Jabnoune et al., 2013), TL (Liu et al., 2018), and LAIR (Wang et al., 2018). The functions of the best-studied plant lncRNAs are listed in Table 1.

lncRNAs can also be classified based on their function, such as a decoy, scaffold, guide, signal, or signal enhancer. Decoys, such as IPS1, delay protein function by mimicking specific regions of the protein’s target (Franco-Zorrilla et al., 2007). Scaffolds help to bring multiple proteins and RNAs together to form functional machineries, and recruit RNAs or proteins to a target region, as in RdDM (Matzke and Mosher, 2014). Signals, such as COLDAIR, are expressed under specific conditions to mediate biological processes (Heo and Sung, 2011). However, a single function model does not always apply to lncRNA function. An lncRNA might exhibit several functions which are usually linked. For example, in RdDM, an lncRNA transcribed by RNA polymerase V can act as a guide for the siRNA-AGO complex to the chromatin target site and as a scaffold for chromatin modifying enzymes and proteins.

An alternative model for classifying lncRNA functions is based on their structural features and the types of interactions they have with their targets, such as DNA interactions or protein interactions (Kung et al., 2013). As in the example of highly complex RdDM pathway, lncRNAs can be expected to have certain secondary structures to bring different chromosome regions or proteins in close proximity. At the end, mechanisms of action include formation of chromosome looping between enhancer and promoter regions, modulation of gene activation and regulation, recruitment of chromatin modifying factors, enhancement of DNA methylation, and chromosome inactivation (Liu et al., 2012).

In some other cases, expression of an lncRNA, rather than the lncRNA itself, is important to initiate a biological process. For example, in mice, rather than the action of lncRNA Airn, its transcription induces Igf2r gene silencing (Latos et al., 2012). Airn is an antisense lncRNA to Igf2r gene whose promoter lies between Airn transcript in the opposite orientation (Santoro et al., 2013). Airn transcribing RNA polymerase prevents assembly of transcription initiation complex at the Igf2r promoter, thus prevents its expression. In another study, mutant lines, of *Arabidopsis*, with an enhanced promoter inside the T-DNA region resulted in a strong expression of a long transcript extending over the promoters of neighboring genes in the same orientation. Similarly, initiation of transcription from an intergenic T-DNA insertion halted expression of a downstream gene in *Arabidopsis* (Hedtke and Grimm, 2009), by making its promoter site inaccessible by transcription initiation complex. In diverse species, polymerase activity extending over the promoter of another gene halted the expression of downstream genes in either opposite or same orientation, indicating that this mechanism is likely to be conserved between species. These studies also emphasize the challenges of functional characterization of lncRNAs.

## Developmental Stage-Related lncRNAs

Many lncRNAs function in developmental pathways in plants. One of the best-characterized examples of this regulation was discovered in *Arabidopsis* at the transition from the vegetative to generative stage. *FLC* is a regulator of flowering time in *Arabidopsis* that represses the induction of flowering (Csorba et al., 2014). An antisense lncRNA to *FLC* gene, COOLAIR, was discovered as upregulated at the beginning of vernalization (Shafiq et al., 2015). COOLAIR is involved in *FLC* repression by both autonomous and vernalization pathways, which leads to flowering in spring. Homology-based search was performed to find FLC locus and antisense FLC transcripts in other monocots, and the results showed that although there is no sequence conservation between antisense FLC transcripts and *Arabidopsis* COOLAIR lncRNA, the locations of these transcripts were conserved in six grass species including *T. aestivum* (Jiao et al., 2019). COLDAIR was identified as another lncRNA potentially regulating *FLC* expression. It is also transcribed in response to cold; however, in contrast to *COOLAIR*, *COLDAIR* is oriented in the sense direction of *FLC* (Heo and Sung, 2011). COLDAIR has been suggested to maintain vernalization by repressing *FLC* (Yamaguchi and Abe, 2012). Both lncRNAs serve as signals that determine the developmental stage of the plant, but much remains to be discovered on their exact functions, interactions and their presence in grass species such as wheat and barley.

Another lncRNA regulating developmental pathways is long-day-specific male-fertility-associated lincRNA (LDMAR). LDMAR expression below a certain level affects pollen development in rice under long-day conditions. Mutations causing reduced expression of LDMAR result in photoperiod-sensitive male sterility in plants grown under long-day conditions (Ding et al., 2012; Zhang and Chen, 2013). Again, the mechanism by which LDMAR regulates pollen development and whether it is expressed in cereals is unclear.

Recently, Guo et al. (2018) identified a novel lncRNA, Wheat Seed Germination Associated RNA (WSGAR), that modulates wheat seed germination. The proposed mechanism of action starts with a wheat-specific miRNA (miR9678) targeting WSGAR, which in turn is processed into phasiRNA and interferes with seed germination. Even though being not well-characterized, another study identified 177 lncRNAs that were responsive to a drug that blocked Ca^2+^ channels in wheat roots. They also observed that lengths of the roots were significantly decreased and root growth was prevented with increasing amounts of drug. Therefore, these 177 lncRNA identified was suggested to be related to root growth in wheat (Ma et al., 2018).

## Stress-Responsive and Other lncRNAs in Wheat, Barley, and Relatives

lncRNAs have been identified in many species from mammals to plants, including model organisms and economically important crop species, as more transcriptomic and genomic data have become available. One of these classes of crops is the Triticeae tribe, which includes cereal species such as wheat and barley important sources of nutrition in the human diet (Moore et al., 1995). Unraveling cellular mechanisms responsible for gene expression under stress conditions is the objective of ongoing research, in efforts to breed cultivars better able to withstand abiotic and biotic stresses (Pieri et al., 2018). For this purpose, the lncRNA repertoires of two of the three diploid wild ancestors of bread wheat (*Triticum aestivum*, AABBDD), *Triticum urartu* (AA) and *Aegilops tauschii* (DD), whose draft and reference genomes were recently published (Jia et al., 2013; Ling et al., 2013; Luo et al., 2017), were examined. Identified lncRNAs, 13,993 lncRNAs from *T. urartu* and 20,338 from *Ae. tauschii*, were also compared to bread wheat and tetraploid wild emmer wheat (*Triticum turgidum* ssp. *dicoccoides*, AABB), a wild subspecies of *T. turgidum* (AABB), the tetraploid ancestor of bread wheat (Pieri et al., 2018). Comparative analyses using RNA sequencing data suggested that the conservation between lncRNA repertoires decreased as the evolutionary distance increased (Pieri et al., 2018). Wild emmer wheat has long been a promising resource for exploration and exploitation of stress responses, due to the remarkable genetic diversity its wild populations retain. Akpinar et al. (2018) predicted lncRNA genes in the *T. turgidum* ssp. *dicoccoides* genome and investigated potential lncRNA-miRNA-mRNA networks. The results of this study revealed 89,623 lncRNAs where 23,713 were identified as potential miRNA targets (Akpinar et al., 2018). Another study identified lncRNAs in two cultivars of wild emmer wheat, Kiziltan and TR39477, and one durum wheat (*T. turgidum* ssp. *durum*, AABB), a domesticated subspecies of *T. turgidum*, revealing 63,773, 61,823, and 43,932 lncRNAs in Kiziltan, TR39477 and durum wheat, respectively. This study reported that 3% of the identified Kiziltan lncRNAs, 6% of the identified TR39477 lncRNAs, and 4% of the durum wheat lncRNAs were differentially expressed in response to drought and called as ‘drought-responsive’ lncRNAs, with most only expressed under drought (Cagirici et al., 2017). Moreover, lncRNAs were identified from the transcriptome of durum wheat cultivar Svevo concurrently with the assembly of its genome. 115,437 lncRNAs were identified and chromosome 3B contained the highest number of lncRNA genes (Maccaferri et al., 2019).

As its ancestors, the bread wheat genome and transcriptome were investigated for lncRNA expression patterns under various biotic and abiotic stress conditions. An analysis of lncRNAs in bread wheat genotypes revealed 77 that were responsive to heat stress, 71 to fungal infection, and 23 to both conditions (Xin et al., 2011). A more comprehensive study identified lncRNAs from 52 sets of RNA sequencing data obtained under heat and drought stress, concluding that 29% of the lncRNAs were responsive to these abiotic stress conditions. Furthermore, the same study explored lncRNA expression under salt stress and identified two lncRNA groups showing distinct expression patterns; one was upregulated in the first hours after exposure and downregulated later, and the second group showed the opposite pattern (Shumayla et al., 2017).

Barley is another economically important species consumed worldwide, and has been studied for a better understanding of response mechanisms to stress (Gozukirmizi and Karlik, 2017). One study examined the barley transcriptome for lncRNAs and their expression patterns under excess boron (Karakulah and Unver, 2017). A second study observed differential expression patterns of two specific lncRNAs in cultivars exposed to salinity; one of those lncRNAs, AK372814, was upregulated under salinity stress (Karlik and Gozukirmizi, 2018), providing a clue to gene regulatory elements involved in responses to salinity. These results give a broad perspective of expression patterns and abundance of lncRNAs in genomes, suggesting that lncRNAs function in cellular mechanisms that are regulated under various stress conditions. However, specific lncRNAs that are involved in stress response pathways largely remain to be identified. Even though next-generation sequencing has provided insight into many species’ genomes and transcriptomes, it will be a long path to narrow down these findings and identify the cellular pathways responsible for stress resistance and regulatory molecules.

## Stress-Responsive lncRNAs in Other Crops

Maize is another important crop and perhaps one of the plant species that has been most extensively studied for lncRNAs. Maize lncRNAs are mostly single exonic and found in intergenic regions, whereas only a small portion coincide with protein-coding genes on the genome (Li L. et al., 2014). Attempts to find lncRNAs responsive to drought revealed 664 lncRNAs that were differentially expressed under drought stress, and were also identified as potential precursors for small non-coding RNA (snRNA) species such as miRNAs, siRNAs, and shRNAs (Zhang et al., 2014). In addition to drought, differentially expressed lncRNAs were predicted in maize under nitrogen deficiency, with most being downregulated. These nitrogen deficiency responsive lncRNAs were examined for co-expression with protein-coding transcripts; 32 were co-expressed with 239 protein-coding transcripts in functional annotation categories including NADPH/NADH dehydrogenation, indicating that these lncRNAs are potential regulators of nitrogen assimilation and photosynthesis since elevated NADH/NADPH consumption is associated with nitrogen assimilation and since photosynthesis reactions are the most important NADPH resources (Lv et al., 2016). In rice (*Oryza sativa*), lncRNAs were investigated under drought and cadmium stress. Under drought stress, 98 lncRNAs were differentially regulated (Chung et al., 2016). Under cadmium stress, 122 of the differentially-expressed transcripts were defined as lncRNAs (He et al., 2015). However, the functions of these lncRNAs are unclear. As in cereals, attempts to discover stress responsive lncRNAs in other crops are still in progress.

## Drawbacks in lncRNA Identification and Target Prediction

Current methods used to identify lncRNAs are not sufficiently accurate or comprehensive. In the absence of a standardized set of selection criteria, researchers must design their own pipelines and decide on the thresholds and tools to use, which may cause incorrect and conflicting results to accumulate in the literature and in databases. Despite continuous efforts to identify lncRNAs from many species, methods developed to date are far from complete, especially due to the complex and unclear nature of these molecules.

In contrast to mRNAs, lncRNAs rarely show evolutionary sequence conservation among species (Ponjavic et al., 2007; Diederichs, 2014). Therefore, instead of directly selecting transcripts that show sequence similarities with lncRNAs of closely related species, lncRNA identification pipelines highly depend on the elimination of RNAs that exhibit mRNA-like and snRNA-like features and classification of the remaining transcripts as lncRNAs. However, the precise identification of the whole lncRNA repertoire for an organism seems impossible due to transcripts that are short and protein coding, and transcripts that are non-coding with long ORFs. Therefore, researchers should also be cautious when considering novel protein-coding transcripts; some transcripts that do not show homology to known sequences stored in public databases might represent undiscovered short protein-coding sequences that could be misannotated as lncRNAs.

Although identifying conserved lncRNA sequences has proven challenging, studies of plant and animal transcriptomes have suggested better sequence conservation at lncRNA promotor sites of vertebrates than the sequence conservation at lncRNA transcripts, particular gene structures and locations around protein-coding genes (Kutter et al., 2012; Johnsson et al., 2014; Nitsche et al., 2015; Deng et al., 2018; Singh et al., 2018), as well as at the structural and functional levels (Kashi et al., 2016). Such positional information and gene structure characteristics such as splice sites will reveal lncRNA genes in other organisms and guide researchers toward more accurate lncRNA identification; however, this approach requires high-quality reference genomes and transcriptomes. Moreover, the features of lncRNAs when folded into secondary and tertiary structures and the relationship between conformation and function suggest another promising opportunity for better lncRNA prediction. However, *in silico* RNA folding algorithms are usually more inaccurate as the transcript length increases (Mathews and Turner, 2006). Even though the relationship between structure and function has been examined for few lncRNAs, studies evaluating the complete folding process of lncRNAs have identified domains that might be important for functional interactions and have compared the folding characteristics of lncRNA with other RNA species computationally (Yang and Zhang, 2014; Liu et al., 2017). Considering that lncRNA secondary and tertiary structures might be important for their interactions and cellular activities (Johnsson et al., 2014) and considering that even lncRNAs that are not conserved can still adopt the same secondary structures (Diederichs, 2014), gaining more information about lncRNA folding might contribute to lncRNA identification by facilitating searches for evolutionary conservation in secondary and tertiary structures instead of in the primary sequences.

The low expression of lncRNAs and expression profiles that are tissue- or developmental stage-specific have further hindered their discovery (Tsoi et al., 2015). Expression profiles of lncRNAs might also provide clues for the prediction of new lncRNAs. However, transcripts with low abundance are usually harder to capture with conventional RNA sequencing applications (Clark et al., 2015; Kashi et al., 2016). Tissue- or developmental stage-specific lncRNAs are also difficult to detect. The time or conditions of sample collection can directly affect which lncRNAs appear in the sequencing results and exclude others expressed at different stages, in different tissues, or under different conditions.

## Future Perspectives and Conclusion

*De novo* assembled partial transcripts used to cause trouble in the identification of any molecule, leading to false annotations or underestimation of transcriptomes. Especially in the case of lncRNAs, these erroneous annotations become very hard to distinguish due to the fact that lncRNAs lack sequence conservation. For that reason, obtaining a well-assembled transcriptome data and having chance to locate the annotated lncRNAs will greatly advance the lncRNA identification procedures.

Now that we have the high-quality reference genomes of wheat and barley, it is now time to use them as efficient as possible. To do that, both breeders, biologists and bioinformaticians should undertake responsibilities and work for better tools and methods. Drawbacks that has been encountered in currently-used lncRNA identification strategies should be overcome for a better understanding of mechanisms lying behind important traits to be used for developing more resistant and more yielding cultivars. Despite the fact that it is challenging, machine learning approaches give promising outcomes in terms of the identification of a group of non-conserved molecules, lncRNAs. Further development of these approaches may lead us to discover other features of lncRNAs that are conserved, such as location, folding characteristics or function. For instance, development of better algorithms that assess folding of lncRNA transcripts would provide clues on their interaction interfaces and thus, on their interacting partners. Similarly, gaining more idea about the interacting partners of a lncRNA would direct us to its function in molecular pathways. Altogether, even though we still have a long way to go until perfectness in lncRNA identification, wheat and barley reference sequences provides a more precise perspective. Better understanding the world of lncRNAs by the help of reference sequences would lead us to the development better cultivars to feed the planet.

## Author Contributions

HB conceived and designed the study. HB, SK, and HC wrote the article.

## Conflict of Interest

The authors declare that the research was conducted in the absence of any commercial or financial relationships that could be construed as a potential conflict of interest.
